# Box rest and analgesia compared to arthroscopic debridement for lame horses with hindlimb subchondral lucencies

**DOI:** 10.18849/ve.v8i2.603

**Published:** 2023-04-19

**Authors:** Charlotte Taylor+, Julie Dubuc+

**Keywords:** SUBCHONDRAL, LUCENCY, CYSTIC, EQUINE, STIFLE, TIBIA, CONDYLE, HORSE, ARTHROSCOPIC DEBRIDEMENT

## Abstract

**PICO question:**

In lame horses, caused by osseous cyst-like lesions in the proximal hindlimb, is box rest and analgesia administration more effective at returning the horse to previous level of performance in comparison to arthroscopic debridement?

**Clinical bottom line:**

**Category of research:**

Treatment.

**Number and type of study designs reviewed:**

Two relevant publications were found, both were retrospective case series.

**Strength of evidence:**

Weak.

**Outcomes reported:**

The success rate of horses returning to previous level of competition following arthroscopic debridement varies widely in the literature available, from 25–86%. While a study reports 64% return to soundness following rest, it is not clear which horses received strict box rest or paddock rest, the duration of the rest period, and whether non-steroidal anti-inflammatory drugs were also prescribed. It is also worth noting that all horses which undergo surgery will also undergo a period of box rest – which makes the two treatment options difficult to compare.

**Conclusion:**

Newer techniques with better success rates are now available and should be considered in lieu of box rest or arthroscopic debridement. Across all treatments available, age remains an important factor with regards to return to soundness, with older horses having a poorer prognosis. Thorough examinations should therefore be performed to rule out concurrent conditions before deciding upon treatment options.

## How to apply this evidence in practice

The application of evidence into practice should take into account multiple factors, not limited to: individual clinical expertise, patient’s circumstances and owners’ values, country, location or clinic where you work, the individual case in front of you, the availability of therapies and resources.

Knowledge Summaries are a resource to help reinforce or inform decision making. They do not override the responsibility or judgement of the practitioner to do what is best for the animal in their care.

## Clinical scenario

You are presented with a 5-year-old recently retired Thoroughbred which is now being retrained for eventing. The owner is concerned that the horse is becoming disunited at canter on the right rein and it has refused to jump yesterday, which it had never done before. Following regional diagnostic anaesthesia, you identify the left stifle as the source of lameness. Indeed, the owner’s complaint under saddle has resolved after blocking the left medial femorotibial joint, while a previous femoropatellar block left the horse unchanged.

## The evidence

The quality of evidence available for this clinical question is low. The authors only found two publications relevant to the question, all were retrospective case series, with no randomised trials comparing treatment with arthroscopic debridement and box rest. The authors found one publication directly comparing the two treatment options (Textor et al., 2001), although all lucencies involved the proximal tibial plateau and only two horses were treated with rest. A more recent publication (Santschi et al., 2020) includes lucencies of the femoral condyles and the proximal tibial plateau but there was only one horse in the arthroscopic debridement group.

## Summary of the evidence

**Textor et al. (2001):** 

Population:	Horses (nine males, three females), mixed breeds, aged 6 months to 12 years old.
Sample size:	12 horses with tibial plateau lucencies: two with bilateral lesions.
Intervention details:	Horses divided in two groups: young (6/12): considered osteochondrosis (mean age 12 months); older (6/12): considered osteoarthritis (OA) (mean age 9 years). Two treatment categories: arthroscopic debridement for lesions in cranial third of the plateau (7/12); other treatments (5/12).
Study design:	Retrospective case series.
Outcome studied:	Lameness.
Main Findings (relevant to PICO question)	Duration of lameness: mean 6.4 months (range: < 1 month to > 2 years). Lameness grade from 0–3 (on 0–5 scale). 7/12 horses (58%): Arthroscopic debridement (four young, three older): of the four young, three performed athletically, one was euthanised; of the three old, one sound, two returned to lower level. 5/12 horses (42%): Other treatments: Horse 1 (young): Stall rest for 8 weeks, anti-inflammatory for 2 weeks, intramuscular pentosan polysulphate for 4 weeks (3 mg/kg, every 7 days). Had surgical debridement at 1 year of age as persistent lameness, in training at 2 years old. Horse 2 (young): Stall rest for 8 weeks, weekly intrasynovial injections of polysulphated glycosaminoglycans in both femorotibial joints (bilateral lucency), had an 8 year racing career with intermittent hindlimb lameness. Horse 3: transcortical debridement open. Euthanised. Horse 4: Non-steroidal anti-inflammatory drugs (NSAIDs). Lost to follow-up. Horse 5: no treatment. Euthanised.
Limitations:	Follow-up information hard to follow. Outcome is unclear as sometimes presented as soundness versus return to exercise. Management of all five horses in other treatment group was different. No cases of femoral lucencies.

**Santschi et al. (2020):** 

Population:	Horses (seven females, 10 males, aged 4 months to 15 years, mixed breeds).
Sample size:	17 horses with proximal tibial lucency.
Intervention details:	Stifle radiographs (at least caudoproximal 15°-craniodistal) one of four treatment options: 6/17 horses (35%) – Limited exercise (box rest) or round pen confinement for 90 days + NSAIDS based on lameness (variable amount of time). 1/17 horses (6%) – Arthroscopic debridement. 8/17 horses (47%) – Screw placement transcondylar or tibial. 2/17 horses (12%) – No treatment.
Study design:	Retrospective case series.
Outcome studied:	Radiographic healing. Resultant lameness. Level of exercise reached following procedure.
Main Findings (relevant to PICO question)	All but one were unilateral. 11/17 horses (65%): primary (no other subchondral lucencies [SCL]), all except one in lateral tibial plateau (TP): 9/11 horses (82%) diagnosed < 1 year old. No lameness to lame at the walk. Follow-up for nine primary horses: One euthanasia (debrided arthroscopically, after rest failed). Six horses without lameness (three rest and three screws). Two horses lame broodmares (rest). 6/17 horses (35%): secondary to medial femoral condyle (MFC) SCL, all in medial TP, two bilateral: All ≥ 3 years old. All either lame at trot or walk. All but one had lag screws (tibia only or tibia + MFC). Follow-up for five secondary horses: Three with tibial and MFC screws: lame at flexion or trot only; Two with tibial screws (one had MFC before): one lame at walk and one sound (ranch rodeo horse).
Limitations:	Little information on analgesia treatment (length, NSAIDS used). Only one debridement case, which also had stem cells. Small numbers for rest group. Rest included round pen, not only.

## Appraisal, application and reflection

Subchondral lucencies are areas of reduced bone density on radiographs, often surrounded by sclerosis and usually located over weight-bearing surfaces (Stewart & Reid, 1982). In equine hindlimbs, the medial femoral condyle (MFC) is most commonly affected but lucencies of the proximal tibia have also been described concurrently or not, to femoral lesions (Jeffcott & Kold, 1982; Verschooten & De Moor, 1982; Textor et al., 2001; Bonilla et al., 2016; and Santschi et al., 2020). Histopathologic examinations of lucency linings have revealed an upregulation of inflammatory cytokines, namely IL-1 and IL-6, and an increased production of prostaglandin E2, which laid the foundation for the inflammatory theory (von Rechenberg et al., 2004). The origin of subchondral lucencies is still debated today and has in the past fallen under the osteochondrosis umbrella. Work from Ray et al. (1996) suggested that trauma to the weight-bearing surface could contribute to the development of these lucencies. In addition, more recent work on juvenile equine femoral condyles (Lemirre et al., 2021) failed to identify chondronecrosis, a hallmark of osteochondrosis, within these subchondral lucencies, which reinforces the argument in favour of the traumatic origin for these lesions.

Subchondral lucencies of the proximal hindlimb have historically been treated through rest with or without anti-inflammatories. While a success rate of 64% (16/28) for return to soundness has been reported (Jeffcott & Kold, 1982; and Stewart & Reid, 1982), the number of horses box rested compared to rested in a small paddock or a round pen remains unclear. The duration of the rest period also differs between studies from unknown (Stewart & Reid, 1982; and Verschooten & De Moor, 1982) to 90 days (Santschi et al., 2020), 8 weeks (Textor et al., 2001) and 6 months (Jeffcott & Kold, 1982). There is no established protocol for the use of NSAIDs during box rest or light work with variable dose range, frequency and duration of administration retrieved from the different papers: none (Jeffcott & Kold, 1982), as needed with light work (Stewart & Reid, 1982), unknown (Santschi et al., 2020) or 4.4 mg/kg of phenylbutazone orally during the rest period (Bonilla et al., 2016). While there are recent publications including horses treated with rest, the numbers of horses treated conservatively is always very small and the lack of standardised treatment does not allow any extrapolation of the results. While the oldest publications often include a large number of conservatively treated horses, their outcome is not always presented clearly, often with no difference made between soundness and return to previous level of exercise. In all the studies, soundness is either evaluated by their referring veterinary surgeons or the owners, which could make the follow-up data less reliable. This is important as the degree of lameness in relation to the size of the lucency remains poorly understood. Recently, a longitudinal radiographic follow-up of shallow MFC lucencies (≤ 3 mm) in young racing thoroughbreds showed these can resolve on their own (6.1%; 15/248), decrease in size (23.6%; 59/248), or increase in size (8.2%; 20/248). While 40.7% (101/248) of the lucencies did not change in size and only 3.6% (9/248) developed into a cyst, those horses still had less race starts as 2-year-olds than their maternal siblings free of stifle pathology (Pérez-Nogués et al., 2021).

Surgical management has been historically recommended with presence of lameness, persistent joint effusion and for horses destined to athletic use (Ortved, 2017). The surgical debridement of subchondral lucencies has been developed in order to reduce the inflammation present within the cavity and the arthroscopic technique was first described by Lewis in 1987. It then remained the favored treatment for these lesions for the following 20 years. Four publications were found looking at arthroscopic debridement of the medial femoral condyles and one for proximal tibial plateau lesions. There is no consistent approach to arthroscopic debridement of the lucencies. For example, some authors used osteostixis with a 3.2 mm drill bit within the lucency cavity following its debridement (Howard et al., 1995). Others have enlarged the lucency opening at the level of the joint space to 1 cm diameter (Schneider et al., 1997) possibly to decrease intraosseous or intracystic pressures which has been hypothesised as one cause of pain in these cases (Ortved, 2017). This is an interesting modification of the technique considering that medial meniscal injuries were later recognised following debridement of some medial femoral condyle lucencies (Hendrix et al., 2009). Four of these meniscal lesions were graded 3 according to Walmsley et al. (2003), indicating a tear of the cranial horn of the medial meniscus and its cranial meniscotibial ligament which extends underneath the femoral condyle (caudal limit not visible).

The success rate across the debridement studies varies between 25% and 86% (Schneider et al. (1997). Schneider et al. (1997) described a much better success rate with focal lucencies (86%; 6/7) compared to diffuse ones (25%; 1/4), but all the horses included in that publication had normal radiographic findings pre-operatively. This could be due to radiographic technique or positioning or the fact these 11 cases had only a small defect or flattening in the medial femoral condyle (grade 1: flattening or small defect in the subchondral bone of the central MFC, Santschi et al., 2015). The success rate in the study employing osteostixis was low at 56% (22/39) (Howard et al., 1995) with almost half the horses (44%; 17/39) having had drilling. It is unclear from the results how many of the successful horses had osteostixis compared to the ones which only had debridement. Interestingly, 68% of that horse population was aged 1–3 years old. This is in direct contradiction to several other studies which have reported a better outcome in younger horses with 3 years old advanced as a non-official cut off (Jeffcott & Kold, 1982; Stewart & Reid, 1982; and Smith et al., 2005). Smith et al.’s publication is extremely thorough in terms of follow-up and reported a 69% (27/39%) return to previous level of exercise in horses < 3-years-old compared to 29% (13/45) for horses > 3 years of age, in a population mainly composed of thoroughbreds. It is possible that the added osteostixis is not beneficial to treatment of the subchondral lucency but this cannot be determined with certainty based on this data only. The presence of other anomalies within the femorotibial joint also comes into play with older horses. Indeed, enthesiophytosis of the intercondylar eminence of the proximal tibia (seven horses) and the intercondylar fossa of the distal femur (eight horses) was detected on pre-operative radiographs with more than 70% (5/7) of these being > 3-years-old (Smith et al., 2005). In addition, cartilage lesions other than at the lucency site were noted in 24% (20/85) of horses, 80% (16/20) of which were aged over 3-years-old.

Following the report of concurrent meniscal lesions following lucency debridement (Hendrix et al., 2009) and a 67%  (35/52) success rate following arthroscopic intralesional injection of medial femoral condyle lucencies with corticosteroids (Wallis et al., 2008), the arthroscopic debridement technique fell out of fashion, especially if the subchondral bone plate was considered stable by the surgeon. Interestingly, the return to racing following intralesional corticosteroid administration in a recent study remains unchanged (68%; 21/31 [Klein et al., 2022]) as first described by Wallis et al. in 2008. Newer techniques, such as the transcondylar screw placement across the subchondral lucency have shown more promising results (Santschi et al., 2015; and Ravanetti et al., 2021). The original technique reported a 75% (15/20) return to soundness 120 days after insertion of a single screw across medial femoral condyles lucencies and decreased the lucency size as visible on radiographs (Santschi et al., 2015). That original study comprised 13 racehorses and it was later found that 77% of these went on to race successfully (Santschi, 2021). Most recently, a 73% (16/22) successful return to racing was also reported following insertion of an absorbable implant inserted in medial femoral condyle lucencies (Ravanetti et al., 2021). It should be noted that any surgical procedure will entail a certain amount of box or paddock rest with the administration of analgesia and the contribution of these to lucency healing and lameness remains unknown.

The authors of this Knowledge Summary search failed to find even one single study comparing box rest with arthroscopic debridement including a decent number of cases in each group. The evidence in the literature at present is weak and does not allow recommendation of box rest and analgesia over arthroscopic debridement nor vice versa. The latest return to racing data reported for arthroscopic debridement was 72% (42/57), with no conservative management group in that study either (Klein et al., 2022). There is, however, substantial evidence that other / newer techniques are now available and should be considered as treatment for these lesions possibly in lieu of box rest and arthroscopic debridement. Indeed, reported success rates for return to racing are higher with the transcondylar screw technique (77% return to racing; 10/13 [Santschi, 2021]) and intralesional mesenchymal stem cells (84% raced after surgery; 16/19 [Klein et al., 2022]).

As with other techniques, the age factor (< 3-years-old having better odds) has also been reported with the transcondylar screw technique (Santschi et al., 2015). In the authors opinion, subchondral lucencies in older horses should be evaluated carefully for the presence of other lesions within the femorotibial joints and clinical decision making done taking these factors into consideration, along with response to intra-articular diagnostic analgesia. While return to soundness is a desirable outcome, not regaining soundness does not preclude return to previous level of exercise and follow-up information should be considered carefully in light of this.

## Methodology

**Search Strategy:** 

Databases searched and dates covered:	CAB Abstracts on OVID Platform 1973–Week 08 2023 PubMed on the NCBI interface Platform 1920–February 2023
Search strategy:	CAB Abstracts: (Horse* or equine* or pony or ponies or mare* or colt* or filly or fillies or stallion or racehorse or thoroughbred).mp. or exp horses/ or exp mares/ or exp colts/ or exp stallions/ or exp thoroughbred/ or exp racehorses/ (OCCL or 'osseous cyst-like lesion*' or 'osseous cyst like lesion*' or 'subchondral bone cyst*' or 'subchondral cystic lesion*' or SCL).mp. (Stifle* or 'proximal hindlimb*' or 'femoral condyle*' or 'medial femoral condyle' or MFC or femur or tibia or ('medial condyle' and femur*)).mp. (rest or 'box rest' or (conservative and (management or treatment or approach))).mp. (Arthroscop* or curettage or ((arthroscopic or surgical) and debridement )).mp. 1 and 2 and 3 and (4 or 5)PubMed: Horse OR equine OR pony OR mare OR colt OR filly OR stallion OR racehorse OR thoroughbred OCCL OR 'osseous cyst-like lesion' OR 'osseous cyst like lesion' OR 'subchondral bone cyst' OR 'subchondral cystic lesion' OR SCL Stifle OR 'proximal hindlimb' OR 'femoral condyle' OR 'medial femoral condyle' OR MFC OR femur OR tibia (rest OR 'box rest' OR (conservative AND (management OR treatment OR approach))) Arthroscopy OR curettage OR ((arthroscopic OR surgical) AND debridement) 1 AND 2 AND 3 AND (4 OR 5)
Dates searches performed:	28 Feb 2023

**Exclusion / Inclusion Criteria:** 

Exclusion:	Proceedings, distal limb subchondral lucencies (SCL), other osteochondral lesions, concurrent ostechondritis dissecans (OCD), surgical debridement through open technique or transcortical, other language than French or English, case reports, narrative reviews, opinion pieces.
Inclusion:	Subchondral lucencies of proximal tibia and distal femur.

**Search Outcome:** 

Database	Number of results	Excluded – Proceedings, case report, reviews, book chapters	Excluded – Not published in French of English	Excluded – Not directly relevant to PICO question	Excluded – Duplicate	Excluded – Duplicate from CAB	Total relevant papers
CAB Abstracts	39	6	3	25	3	-	2
PubMed	31	0	0	24	2	5	0
Total relevant papers when duplicates removed							2

## ORCiD

Julia Dubuc: https://orcid.org/0000-0003-0552-9117

## Conflict of Interest

The authors declare no conflicts of interest.
